# The HMG‐CoA Reductase Inhibitor Fluvastatin Promotes Th2‐Skewed Immune Responses by Modulating Dendritic Cell Differentiation

**DOI:** 10.1111/gtc.70148

**Published:** 2026-09-01

**Authors:** Satoshi Ishikawa, Haruka Mizuno, Kanon Murase, Kazuki Sasano, Isamu Ogawa, Yuka Itoh, Saotomo Itoh, Shigeaki Hida

**Affiliations:** ^1^ Department of Molecular and Cellular Health Sciences Graduate School of Pharmaceutical Sciences, Nagoya City University Nagoya Japan; ^2^ Laboratory of Hygienic Chemistry School of Pharmacy, Aichi Gakuin University Nagoya Japan

## Abstract

Statins, inhibitors of HMG‐CoA reductase, exert immunomodulatory effects beyond lipid lowering, yet how they regulate T helper (Th) cell differentiation remains poorly understood. We show that fluvastatin administration to C57BL/6 mice for 7 days suppressed interferon‐γ and elevated interleukin‐4 production by splenic CD4^+^ T cells, shifting the immune balance toward Th2 without altering splenocyte numbers or major immune cell subset proportions. This effect was not shared by rosuvastatin, suggesting that lipophilicity‐dependent cellular uptake influences immunomodulatory potency. In vitro experiments confirmed a direct Th2‐promoting action of fluvastatin on CD4^+^ T cells. Basophils were excluded as mediating cells, as fluvastatin suppressed rather than enhanced basophil interleukin‐4 production. Continuous fluvastatin exposure during granulocyte‐macrophage colony‐stimulating factor (GM‐CSF)‐driven dendritic cell (DC) differentiation altered the phenotype of the resulting DCs and markedly enhanced their Th2‐polarizing capacity in co‐cultures with antigen‐specific CD4^+^ T cells. These functional changes were accompanied by alterations in DC surface molecule expression, although their causal contribution remains to be established. These findings indicate that continuous fluvastatin exposure during DC differentiation is associated with an altered DC phenotype and enhanced Th2‐polarizing capacity.

## Graphical abstract

Fluvastatin promotes Th2‐skewed immune responses through direct effects on CD4^+^ T cells and, importantly, by altering dendritic cells (DCs) during differentiation. Continuous exposure at a near‐physiological concentration modifies DC phenotype and enhances their subsequent Th2‐polarizing capacity.

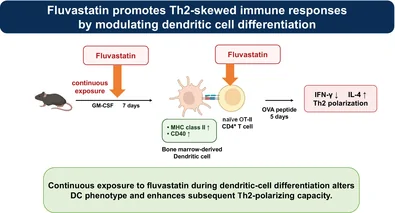

## 1 Introduction

Dyslipidemia, characterized by abnormal blood lipid levels, is a prevalent lifestyle‐related disorder and a major risk factor for atherosclerosis, ischemic heart disease, and cerebrovascular disease. Its global prevalence continues to rise in parallel with population aging and shifts in dietary habits (Cholesterol Treatment Trialists Collaboration et al. 2010; Grundy et al. 2019). Among the lipid parameters implicated in cardiovascular risk, low‐density lipoprotein cholesterol (LDL‐C) plays a central role in atherogenesis, making its management essential for cardiovascular disease prevention. HMG‐CoA reductase inhibitors (statins) lower LDL‐C by competitively inhibiting cholesterol biosynthesis, and their efficacy in reducing cardiovascular events has been established across numerous large‐scale clinical trials, cementing their status as the first‐line pharmacotherapy for dyslipidemia.

Beyond their lipid‐lowering effects, statins exert pleiotropic actions that include improvement of vascular endothelial function, attenuation of oxidative stress, inhibition of thrombogenesis, and modulation of inflammatory and immune responses. These effects are not merely secondary to cholesterol reduction; rather, they are thought to arise from alterations in intracellular signaling caused by inhibition of the mevalonate pathway. This pathway governs not only cholesterol synthesis but also the production of isoprenoid intermediates—farnesyl pyrophosphate (FPP) and geranylgeranyl pyrophosphate (GGPP)—which are required for the prenylation of small GTPases such as Ras, Rho, and Rac. Because these GTPases regulate cell proliferation, differentiation, survival, and cytokine production, statin‐mediated disruption of their prenylation has the potential to broadly alter immune cell signaling, differentiation, and function (Liao 2002; Greenwood et al. 2006; Zeiser 2018).

Consistent with this notion, statins have been shown to act on multiple immune cell types, including dendritic cells (DC), macrophages, T cells, B cells, and mast cells. In dendritic cells, statins downregulate MHC class II expression and co‐stimulatory molecule presentation while suppressing inflammatory cytokine production (Kwak et al. 2000; Sun and Fernandes 2003). In T cells, statins inhibit proliferation and modulate cytokine secretion (Youssef et al. 2002; Fehr et al. 2004; Coward and Chow 2006; Jameel et al. 2013), thereby influencing adaptive immune responses. Notably, statins ameliorate disease progression in experimental models of multiple sclerosis and arthritis. In mast cells, they suppress IgE‐mediated degranulation and cytokine release (Kolawole et al. 2016).

Helper T (Th) cells are central orchestrators of the adaptive immune response, differentiating into functionally distinct subsets—Th1, Th2, Th17, and Treg—defined by their cytokine profiles. Th1 cells produce IFN‐γ and drive cellular immunity, whereas Th2 cells secrete IL‐4, IL‐5, and IL‐13 to promote humoral immunity and allergic responses. Dysregulation of the Th1/Th2 balance underlies the pathogenesis of autoimmune, infectious, and allergic diseases. Although statins have been reported to suppress Th1 responses and promote Th2 responses (Youssef et al. 2002; Fehr et al. 2004; Coward and Chow 2006), the findings are inconsistent across studies, likely reflecting differences in statin type, dosage, treatment duration, and the specific immune cell populations examined. The precise molecular mechanisms by which statins reshape the Th balance through actions on particular cell types therefore remain poorly understood.

We have previously demonstrated that cytokines produced by innate immune cells—including dendritic cells (DCs) and basophils—and the transcription factors governing their expression critically regulate Th cell differentiation and the Th2 response. In particular, our group has established that IRF1 and IRF2 are key determinants of the Th1/Th2 balance (Taki et al. 1997; Ichikawa et al. 2004; Hida et al. 2005, 2009). Whether drugs targeting lipid metabolism pathways regulate Th cell differentiation through analogous immunoregulatory mechanisms, however, has not been investigated.

To address this question, we focused on fluvastatin and examined its effects on Th differentiation using both in vivo and in vitro experimental systems. By analyzing T cells, basophils, and DCs, we sought to identify the cellular targets and mechanistic basis of fluvastatin‐induced Th2 polarization. An important consideration in the study design is that much prior research has relied on short‐term exposure to relatively high statin concentrations, which may not reflect clinically relevant exposure during long‐term therapy. Because statins are administered continuously over months to years, progenitor cells—not only mature immune cells—may be persistently exposed to the drug, with potential consequences for immune cell differentiation. Accordingly, the aim of the present study was not simply to confirm the previously reported Th2‐skewing effect of statins, but to determine whether continuous exposure to fluvastatin at a near‐physiological concentration during immune cell differentiation alters subsequent cellular function. By comparing its effects on T cells, basophils, and DCs, we specifically examined whether exposure during the DC differentiation period modifies the phenotype and Th2‐polarizing capacity of the resulting DCs. Based on the reported peak plasma concentration of fluvastatin following standard therapeutic dosing in humans (*C*_max_ 0.3–0.5 μM at 20–40 mg/day), we selected 0.5 μM as the primary in vitro concentration, a level substantially lower than the supratherapeutic concentrations (5–20 μM) commonly employed in previous immunological studies (Tse et al. 1992; Scripture and Pieper 2001; Sun and Fernandes 2003; Jameel et al. 2013).

## 2 Results

### 2.1 Fluvastatin and Lovastatin Shift the Splenic Th1/Th2 Cytokine Balance Toward Th2 In Vivo

**FIGURE 1:**
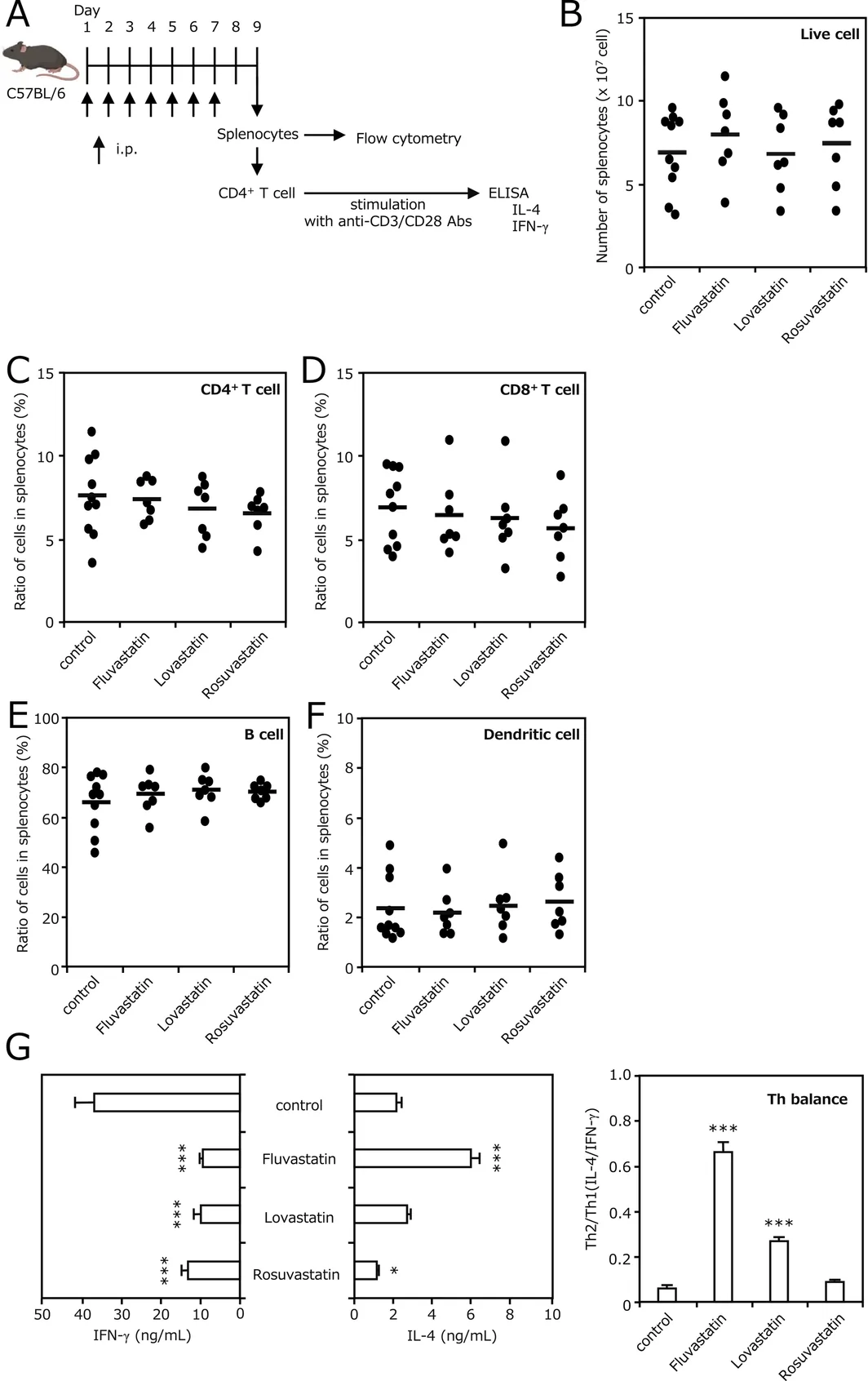
Fluvastatin and lovastatin shift the splenic Th1/Th2 cytokine balance toward Th2 in vivo. (A) Experimental schematic. C57BL/6 mice received daily intraperitoneal injections of fluvastatin, lovastatin, or rosuvastatin (10 mg/kg/day) for seven consecutive days and were sacrificed on day 8. Splenocytes were analyzed by flow cytometry, and splenic CD4^+^ T cells were isolated and stimulated with plate‐bound anti‐CD3 and soluble anti‐CD28 antibodies for cytokine measurement by ELISA. (B) Total numbers of live splenocytes. (C–F) Proportions of CD4^+^ T cells (C), CD8^+^ T cells (D), B cells (E), and DCs (F) among total splenocytes. Data in (B–F) are shown as individual values with the median line; *n* = 7–10 mice per group. (G) IFN‐γ and IL‐4 production by splenic CD4^+^ T cells following anti‐CD3/CD28 stimulation, and the Th2/Th1 ratio (IL‐4/IFN‐γ; right). Data in (G) are representative of four independent experiments; values represent the mean ± SD of triplicate wells within a single representative experiment. *p < 0.05, ***p < 0.001 vs. control (Dunnett's test).

To examine the effects of HMG‐CoA reductase inhibitors on the adaptive immune response, C57BL/6 mice were administered fluvastatin, lovastatin, or rosuvastatin intraperitoneally for seven consecutive days at a dose of 10 mg/kg/day, after which splenocytes were collected and analyzed (Figure 1A).

We first assessed whether drug administration altered immune cell viability or composition. Total viable cell numbers in the spleen did not differ significantly among groups (Figure 1B). Flow cytometric analysis likewise revealed no significant changes in the proportions of CD4^+^ T cells, CD8^+^ T cells, B cells, or DCs (Figure 1C–F). These findings indicate that statin administration under these experimental conditions did not significantly affect the viability or overall composition of major splenic immune cell populations.

To evaluate functional changes in CD4^+^ T cells, splenocytes were enriched for CD4^+^ T cells and stimulated with anti‐CD3 and anti‐CD28 antibodies, and cytokine production was measured. IFN‐γ production was significantly reduced in all three statin‐treated groups (Figure 1G). In contrast, IL‐4 production was markedly elevated in the fluvastatin group, with a trend toward increased production also observed in the lovastatin group (Figure 1G). These results demonstrate that fluvastatin suppresses Th1 responses while promoting Th2 responses in vivo, thereby shifting the immune balance toward a Th2‐dominant state. Notably, this effect was not shared by all statins tested, and distinct patterns were observed for each compound. Because fluvastatin exhibited the strongest Th2‐inducing effect, subsequent experiments focused on elucidating its mechanism of action.

The absence of significant changes in total splenocyte numbers or major immune cell proportions under the dosing regimen used suggests that the observed Th balance shift reflects an alteration in immune function per se, rather than a secondary consequence of cell death or impaired proliferation.

### 2.2 Fluvastatin Directly Enhances IL‐4 Production and Suppresses IFN‐γ Production by CD4 ^+^ T Cells In Vitro

**FIGURE 2:**
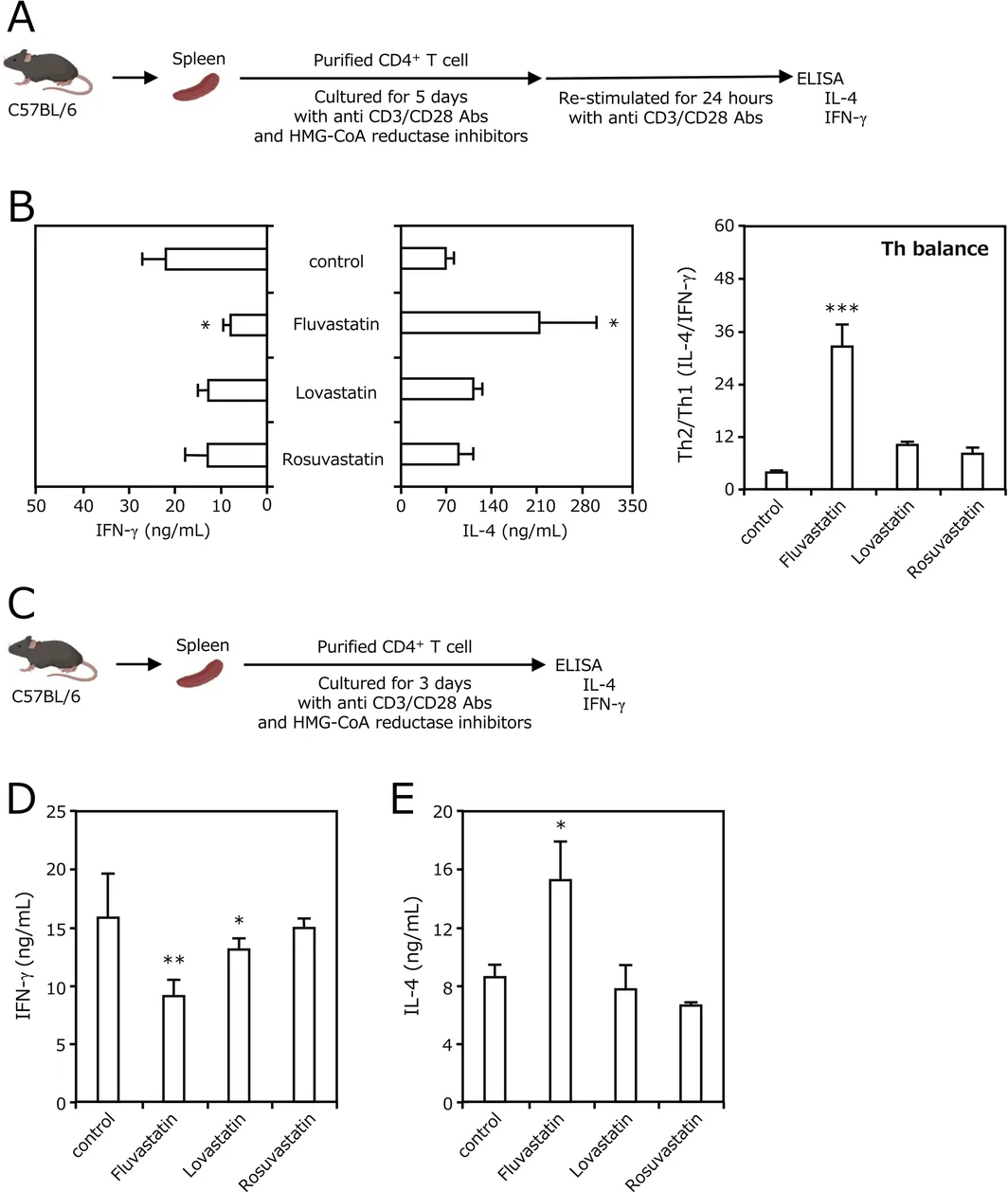
Fluvastatin directly enhances IL‐4 production and suppresses IFN‐γ production by CD4^+^ T cells in vitro. (A) Experimental schematic for the 5‐day differentiation assay. Purified splenic CD4^+^ T cells from C57BL/6 mice were cultured for 5 days with plate‐bound anti‐CD3 and soluble anti‐CD28 antibodies in the presence or absence of HMG‐CoA reductase inhibitors (0.5 μM), then restimulated for 24 h with anti‐CD3/CD28 antibodies, and supernatants were collected for cytokine measurement by ELISA. (B) IFN‐γ and IL‐4 production by splenic CD4^+^ T cells following anti‐CD3/CD28 stimulation, and the Th2/Th1 ratio (IL‐4/IFN‐γ; right), following 5‐day culture and restimulation. (C) Experimental schematic for the 3‐day stimulation assay. CD4^+^ T cells were cultured under the same conditions for 3 days, and supernatants were collected directly for cytokine measurement. (D, E) IFN‐γ (D) and IL‐4 (E) production following 3‐day culture. Data in (B, D, E) are representative of three independent experiments; values represent the mean ± SD of triplicate wells within a single representative experiment. *p < 0.05, **p < 0.01, ***p < 0.001 vs. control (Dunnett's test).

To determine whether the Th2‐skewed response observed in Figure 1 reflected a direct effect on T cells, an in vitro stimulation assay was performed using CD4^+^ T cells isolated from the spleen (Figure 2A). Purified CD4^+^ T cells were cultured for 5 days with anti‐CD3 and anti‐CD28 antibodies, followed by restimulation and cytokine measurement. IFN‐γ production was significantly reduced in the fluvastatin‐treated group, whereas IL‐4 production was markedly increased (Figure 2B).

To further assess effects on the early activation phase, the culture period was shortened to 3 days (Figure 2C). Under these conditions, IFN‐γ production was reduced by both fluvastatin and lovastatin (Figure 2D), while IL‐4 production was significantly increased in the fluvastatin group (Figure 2E).

Taken together, these results demonstrate that fluvastatin acts directly on CD4^+^ T cells to promote Th2 differentiation by upregulating IL‐4 production. The observation that this effect was apparent even under short‐term culture conditions suggests that fluvastatin influences the cytokine production program from the earliest stages of T cell activation. These findings are consistent with previous reports demonstrating that statins modulate cytokine production by CD4^+^ T cells (Youssef et al. 2002; Fehr et al. 2004; Coward and Chow 2006; Jameel et al. 2013).

### 2.3 Basophils Do Not Participate in Fluvastatin‐Mediated Th2 Induction

**FIGURE 3:**
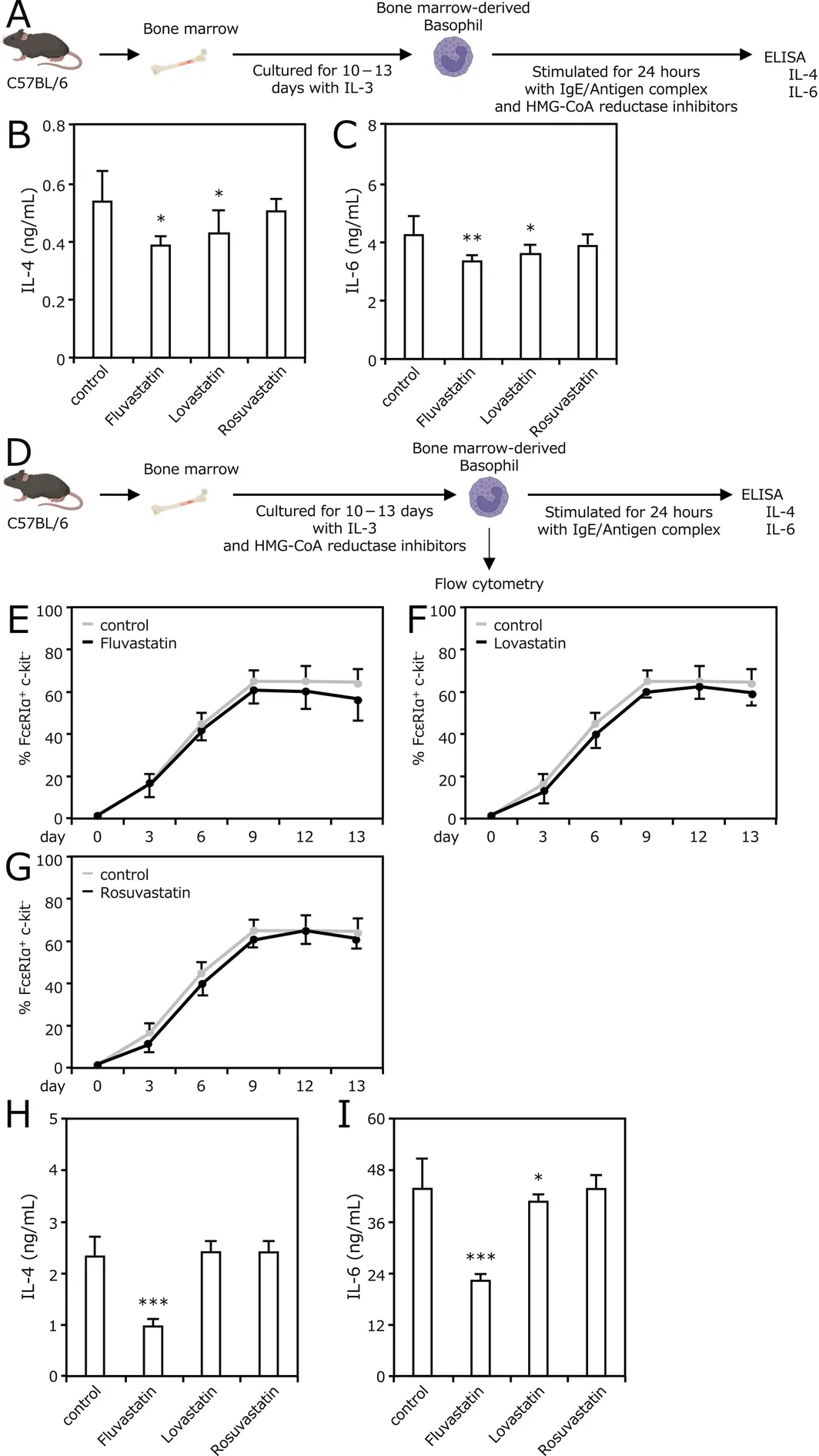
Basophils do not participate in fluvastatin‐mediated Th2 induction. (A) Experimental schematic. Bone marrow–derived basophils (BMBs) were generated by 10–13 day culture with IL‐3 and subsequently stimulated for 24 h with an IgE–antigen complex in the presence or absence of HMG‐CoA reductase inhibitors (0.5 μM). (B, C) IL‐4 (B) and IL‐6 (C) production by BMBs following IgE–antigen complex stimulation. (D) Experimental schematic for the differentiation assay. Bone marrow cells were cultured for 10–13 days with IL‐3 in the continuous presence of HMG‐CoA reductase inhibitors (0.5 μM). (E–G) Proportion of FcεRIα^+^ c‐Kit^−^ cells during differentiation in the presence of fluvastatin (E), lovastatin (F), or rosuvastatin (G). The vehicle control (DMSO) and the three statin‐treated groups were cultured in parallel within the same experiment, and the same vehicle‐control group is shown in panels E–G. (H, I) IL‐4 (H) and IL‐6 (I) production by BMBs differentiated in the presence of inhibitors, following stimulation with an IgE–antigen complex. Data in (B, C, H, I) are representative of three independent experiments; values represent the mean ± SD of triplicate wells within a single representative experiment. *p < 0.05, **p < 0.01, ***p < 0.001 vs. control (Dunnett's test).

We have previously identified basophils as early IL‐4‐producing cells that play a critical role in Th2 immune responses (Hida et al. 2005, 2009; Yoshimoto 2018). We therefore investigated whether fluvastatin modulates IL‐4 production by basophils (Figure 3A).

Bone marrow–derived basophils were stimulated with an IgE–antigen complex. Both fluvastatin and lovastatin significantly suppressed IL‐4 production (Figure 3B), and a comparable inhibitory effect was observed for IL‐6 (Figure 3C).

We next examined whether statins affect basophil differentiation itself. Bone marrow cells were cultured with IL‐3 in the continuous presence of each statin to induce basophil differentiation (Figure 3D). The proportion of FcεRIα^+^ c‐Kit^−^ cells was nearly identical across all groups, indicating that fluvastatin, lovastatin, and rosuvastatin had no significant effect on basophil differentiation (Figure 3E–G). Upon stimulation of these differentiated basophils, fluvastatin significantly suppressed both IL‐4 and IL‐6 production (Figure 3H,I).

These results indicate that fluvastatin suppresses rather than promotes IL‐4 production by basophils. The Th2‐dominant state observed in Figures 1 and 2 is therefore unlikely to be mediated through a basophil‐dependent mechanism and instead reflects the involvement of other immune cell populations.

### 2.4 Fluvastatin Modulates Cytokine Production and Surface Molecule Expression of Bone Marrow–Derived Dendritic Cells

**FIGURE 4:**
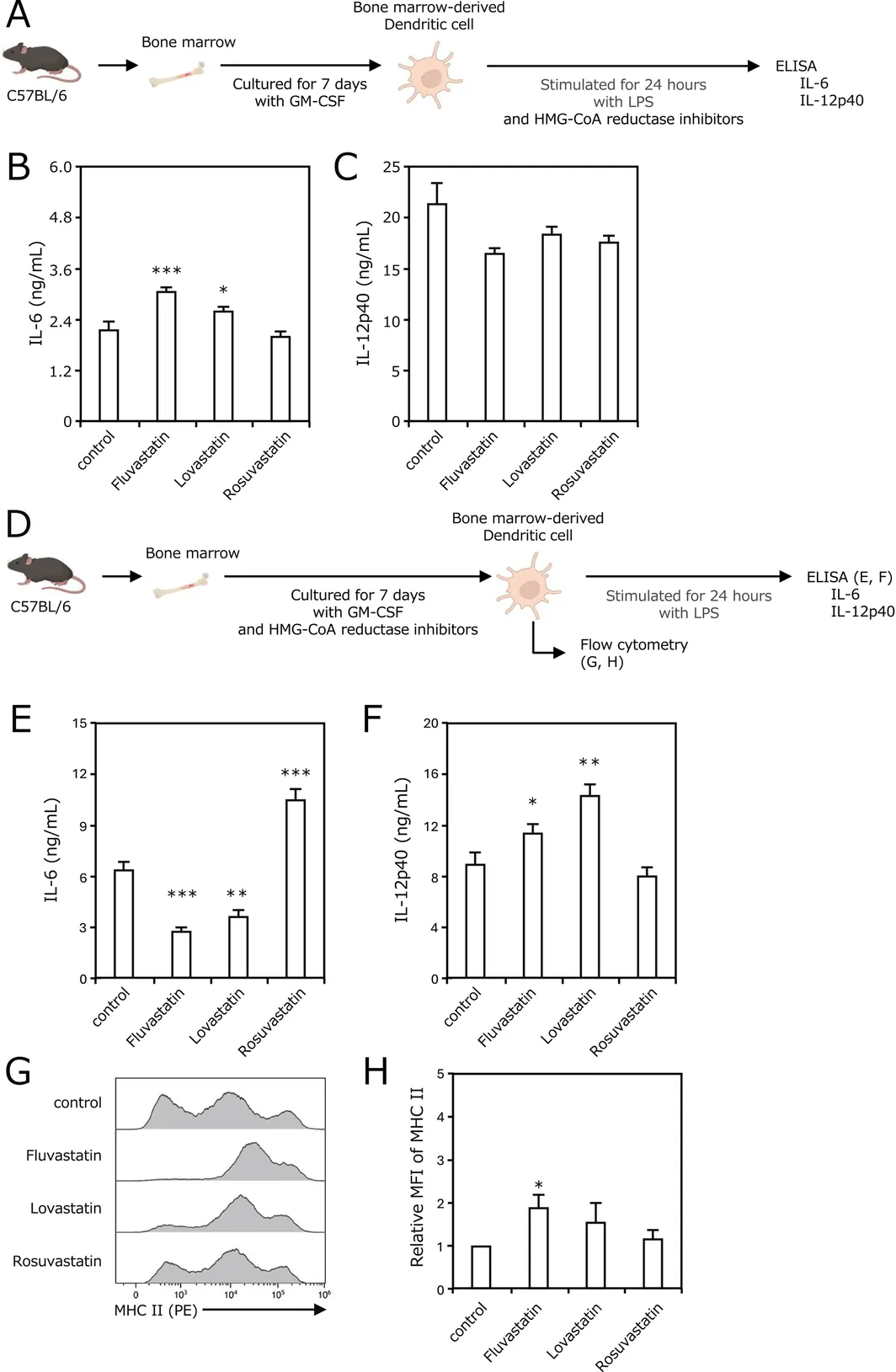
Fluvastatin modulates cytokine production and surface molecule expression of BMDCs. (A) Experimental schematic for the mature BMDC assay. BMDCs were generated by 7‐day culture with GM‐CSF and subsequently stimulated with LPS (100 ng/mL) for 24 h in the presence or absence of HMG‐CoA reductase inhibitors (0.5 μM). (B, C) IL‐6 (B) and IL‐12p40 (C) production by mature BMDCs. (D) Experimental schematic for the BMDC differentiation assay. Bone marrow cells were cultured with GM‐CSF in the continuous presence of HMG‐CoA reductase inhibitors (0.5 μM) for 7 days. At the end of the differentiation period, an aliquot of the resulting BMDCs was analyzed by flow cytometry for MHC class II expression before LPS stimulation. The remaining BMDCs were stimulated with LPS (100 ng/mL) for 24 h for cytokine measurement. (E, F) IL‐6 (E) and IL‐12p40 (F) production following LPS stimulation. (G) Representative flow cytometry histograms of MHC class II (I‐Aᵇ) expression on BMDCs at the end of the 7‐day differentiation period before LPS stimulation. (H) Relative mean fluorescence intensity (MFI) of MHC class II to the control group. Data in (B, C, E, F, H) are representative of three independent experiments; values represent the mean ± SD of triplicate wells within a single representative experiment. *p < 0.05, **p < 0.01, ***p < 0.001 vs. control (Dunnett's test).

Given that DCs are the principal antigen‐presenting cells governing the differentiation of naïve T cells, we next examined the effects of fluvastatin on DC biology (Figure 4A). To evaluate the effects of statins on cytokine production by already‐differentiated DCs, fully differentiated were stimulated with lipopolysaccharide (LPS; 100 ng/mL) for 24 h in the presence or absence of each statin. Fluvastatin significantly increased IL‐6 production (Figure 4B), whereas IL‐12p40 production remained unchanged (Figure 4C).

To investigate the effects of statin exposure during the differentiation process, each statin was added continuously during the 7‐day culture of bone marrow cells in the presence of GM‐CSF (Figure 4D). At the end of the differentiation period and before LPS stimulation, MHC class II expression was assessed by flow cytometry. MHC class II expression was significantly increased in the fluvastatin group, whereas lovastatin and rosuvastatin produced no significant change (Figure 4G,H). The resulting BMDCs were separately stimulated with LPS for 24 h to evaluate cytokine production. Under these conditions, fluvastatin and lovastatin significantly decreased IL‐6 production, whereas rosuvastatin significantly increased IL‐6 production (Figure 4E). Fluvastatin and lovastatin significantly increased IL‐12p40 production, whereas rosuvastatin produced no significant change (Figure 4F).

Notably, the experimental conditions used to examine already‐differentiated BMDCs and differentiating bone marrow cells differed in both the duration of statin exposure and the differentiation state of the cells at the time of exposure. Therefore, the present study cannot determine whether the distinct responses observed under these conditions reflect exposure duration, cellular differentiation state, or both. Together, these findings indicate that statin exposure during DC differentiation and exposure of already‐differentiated BMDCs are associated with distinct cytokine and phenotypic responses.

### 2.5 Dendritic Cells Differentiated in the Presence of Fluvastatin or Lovastatin Exhibit Enhanced Th2‐Polarizing Capacity

**FIGURE 5:**
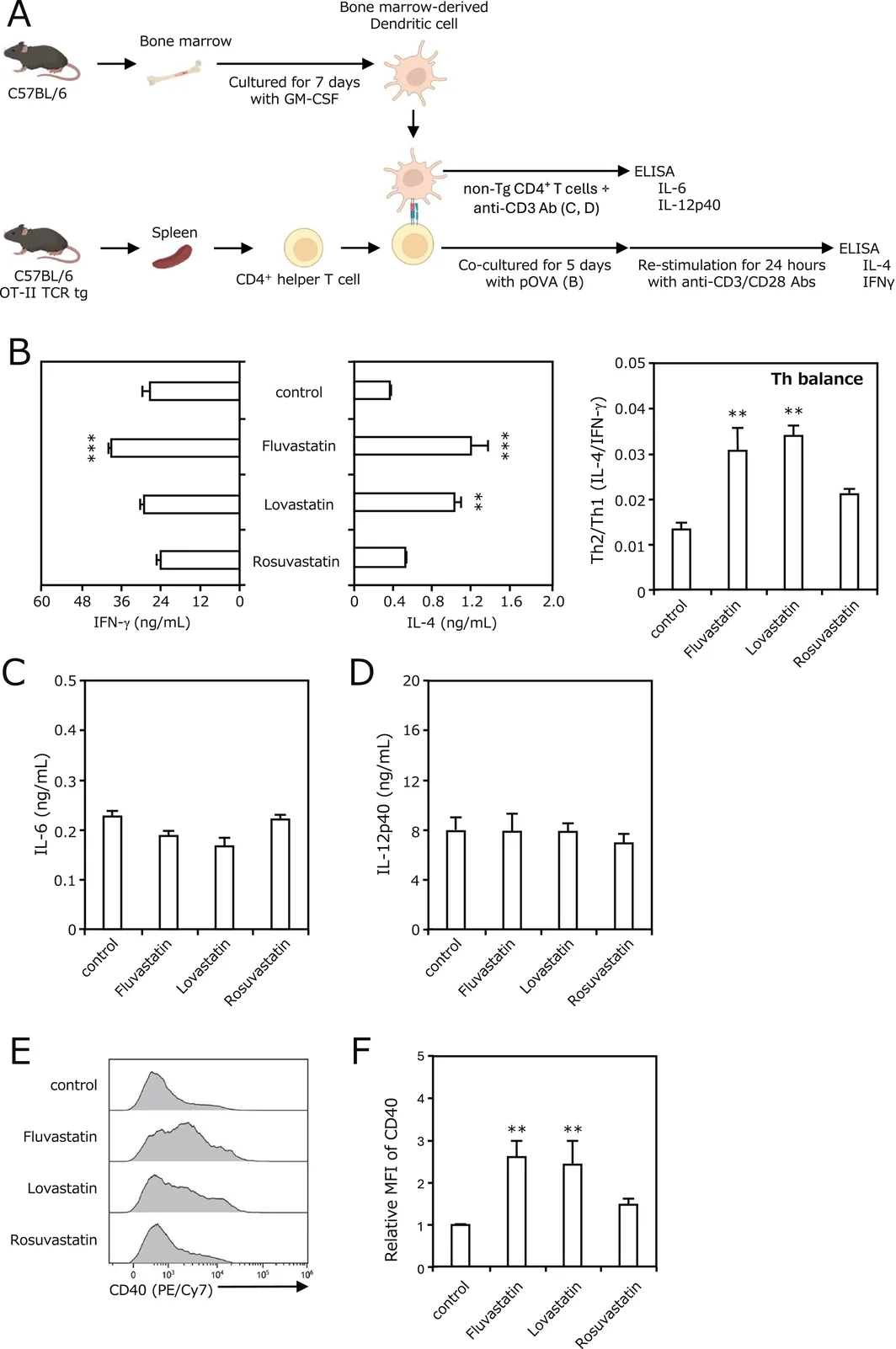
DCs differentiated in the presence of fluvastatin or lovastatin exhibit enhanced Th2‐polarizing capacity. (A) Experimental schematic. BMDCs were generated by culturing bone marrow cells for 7 days with GM‐CSF in the presence or absence of HMG‐CoA reductase inhibitors (0.5 μM). Two separate DC–T cell co‐culture assays were subsequently performed. For the experiments shown in (C) and (D), BMDCs (1 × 10^5^ cells) were co‐cultured with CD4^+^ T cells (1 × 10^6^ cells) from non‐transgenic C57BL/6 mice in the presence of anti‐CD3 antibody (1 μg/mL) for 24 h. Culture supernatants were collected, and IL‐6 and IL‐12p40 concentrations were measured by ELISA. For the Th2‐polarization assay shown in (B), BMDCs were co‐cultured with purified CD4^+^ T cells from OT‐II TCR‐transgenic mice in the presence of OVA_323–339_ peptide (0.5 μM) for 5 days. T cells were subsequently harvested and restimulated for 24 h with anti‐CD3 and anti‐CD28 antibodies, and IFN‐γ and IL‐4 concentrations were measured by ELISA. (B) IFN‐γ and IL‐4 production by CD4^+^ T cells following restimulation, and the Th2/Th1 ratio (IL‐4/IFN‐γ; right). (C, D) IL‐6 (C) and IL‐12p40 (D) concentrations in supernatants from 24‐h co‐cultures of BMDCs and CD4^+^ T cells from non‐transgenic C57BL/6 mice stimulated with anti‐CD3 antibody (1 μg/mL). (E) Representative flow cytometry histograms of CD40 expression on BMDCs assessed at the end of the 7‐day differentiation period before any subsequent stimulation. (F) Relative MFI of CD40 expression to the control group. Data in (B, C, D, F) are representative of three independent experiments; values represent the mean ± SD of triplicate wells within a single representative experiment. **p < 0.01, ***p < 0.001 vs. control (Dunnett's test).

Finally, we investigated whether the fluvastatin‐induced changes in DCs functionally affect T cell differentiation (Figure 5A). BMDCs differentiated in the presence of statins were co‐cultured with CD4^+^ T cells from OT‐II TCR transgenic mice, which express an ovalbumin‐specific T cell receptor. In both the fluvastatin and lovastatin groups, IFN‐γ production was reduced and IL‐4 production was significantly elevated (Figure 5B), resulting in a clear shift of the Th1/Th2 balance toward Th2.

In a separate co‐culture assay, BMDCs differentiated in the presence or absence of statins were co‐cultured with CD4^+^ T cells from non‐transgenic C57BL/6 mice in the presence of anti‐CD3 antibody (1 μg/mL) for 24 h. Under these conditions, no significant differences in IL‐6 or IL‐12p40 production were observed among the groups (Figure 5C,D). Analysis of DC surface molecule expression revealed a significant increase in CD40 expression in both the fluvastatin and lovastatin groups (Figure 5E,F).

Collectively, these findings demonstrate that DCs differentiated in the presence of fluvastatin or lovastatin acquire enhanced Th2‐polarizing capacity. At the end of the 7‐day differentiation period and before subsequent stimulation, CD40 expression was significantly increased in BMDCs generated in the presence of fluvastatin or lovastatin (Figure 5E,F). However, whether this change contributes causally to the enhanced Th2‐inducing capacity remains unknown.

## 3 Discussion

The present study demonstrates that fluvastatin, an HMG‐CoA reductase inhibitor, promotes Th2 differentiation of CD4^+^ T cells through both direct action on T cells and an indirect mechanism mediated by alterations in DC differentiation. The principal novelty of the present study lies not in demonstrating statin‐induced Th2 skewing per se, which has been reported previously, but in showing that continuous exposure to fluvastatin at a near‐physiological concentration during DC differentiation modifies the phenotype and subsequent Th2‐polarizing capacity of the resulting DCs. The immunomodulatory properties of statins have been extensively studied; however, much of the existing literature relies on concentrations of 5–20 μM or higher, at which nonspecific effects such as impaired cell proliferation or cytotoxicity cannot be excluded. Moreover, the majority of studies have examined short‐term responses in already‐differentiated immune cells, leaving the effects of statins on the immune cell differentiation process largely unexplored (Tse et al. 1992; Kwak et al. 2000; Scripture and Pieper 2001; Sun and Fernandes 2003; Jameel et al. 2013).

The present study addressed these limitations by employing fluvastatin at near‐physiological concentrations under conditions that produced no significant changes in cell viability or the composition of major immune cell populations. The observed shift in Th balance in the absence of any detectable cytotoxic effect suggests that fluvastatin modulates immune cell function under these conditions, rather than exerting secondary effects attributable to cell death.

This enhanced Th2‐polarizing capacity was accompanied by several phenotypic changes in the resulting BMDCs. Phenotypic analysis of BMDCs at the end of the 7‐day differentiation period, before subsequent stimulation, revealed increased MHC class II expression in the fluvastatin group and increased CD40 expression in the fluvastatin and lovastatin groups. Thus, both surface molecules were evaluated at a comparable pre‐stimulation stage. Nevertheless, because their expression patterns differed among statins and no functional blocking experiments were performed, the present data do not establish coordinated regulation of MHC class II and CD40 or a causal role for either molecule in the enhanced Th2‐polarizing capacity. Short‐term fluvastatin treatment of already‐differentiated BMDCs increased IL‐6 production (Figure 4B), whereas BMDCs generated in the presence of fluvastatin showed a different IL‐6 response after subsequent LPS stimulation (Figure 4E). However, the Th2‐polarizing capacity was evaluated only for DCs generated in the presence of statins during differentiation (Figure 5B); therefore, the present study does not allow a direct comparison of Th2‐inducing capacity between these two exposure paradigms. Importantly, DCs generated in the presence of fluvastatin or lovastatin exhibited enhanced Th2‐polarizing capacity, indicating that changes acquired under this differentiation‐exposure condition have functional consequences for subsequent T‐cell responses.

Because statins are administered chronically in clinical practice, developing immune cells may also be exposed to these drugs in vivo. Our differentiation‐exposure model captures one aspect of such exposure; however, it does not reproduce human pharmacokinetics and cannot distinguish the effects of exposure duration from those of cellular differentiation state. Our differentiation‐exposure model therefore provides a complementary experimental setting that captures one aspect of chronic statin exposure not addressed by conventional short‐term stimulation protocols (Tse et al. 1992; Scripture and Pieper 2001; Climent et al. 2021).

DCs regulate Th cell polarization through antigen presentation, cytokine production, and co‐stimulatory signaling (Moser and Murphy 2000; Na et al. 2016; Han et al. 2022). CD40 provides an important co‐stimulatory signal during DC–T‐cell interactions (Cella et al. 1996). In the present study, phenotypic changes in MHC class II and CD40 expression were observed in statin‐conditioned BMDCs at the pre‐stimulation stage. Separately, in the 24‐h BMDC–CD4^+^ T‐cell co‐culture assay, IL‐6 and IL‐12p40 production did not differ significantly among the groups. These observations identify several statin‐associated phenotypic and functional changes, but do not establish which, if any, contributes causally to the enhanced Th2‐polarizing capacity. This pattern raises the possibility that alterations in co‐stimulatory molecule expression, including CD40, may contribute to Th2 skewing independently of cytokine‐mediated mechanisms, although a causal role for any individual molecule remains to be established.

Several molecular mediators of DC–driven Th2 induction have been identified in recent years, including OX40L, Jagged1, and IRF4 (Ito et al. 2005; Liotta et al. 2008; Gao et al. 2013). Whether the CD40 upregulation observed here is one such contributing factor, or merely a marker of altered DC maturation state, cannot be determined from the present data. Future functional experiments using CD40 blockade or genetic approaches will be required to determine whether the increase in CD40 expression directly contributes to the enhanced Th2‐polarizing capacity. Comprehensive transcriptomic analysis of fluvastatin‐conditioned DCs will be required to fully delineate the molecular mechanisms underlying their enhanced Th2‐inducing capacity. A further limitation of the present study is that phenotypic characterization of BMDCs was based on a limited set of surface markers. Additional analyses including CD80, CD86, and other differentiation and maturation markers will be required to more comprehensively determine how statin exposure during differentiation affects the resulting DC phenotype.

The molecular mechanisms underlying the phenotypic and functional changes acquired by DCs during fluvastatin exposure remain to be fully elucidated. Statins inhibit the mevalonate pathway, thereby suppressing not only cholesterol synthesis but also the production of farnesyl pyrophosphate (FPP) and geranylgeranyl pyrophosphate (GGPP)—isoprenoid intermediates essential for the prenylation of small GTPases such as Ras, Rho, and Rac, which regulate DC differentiation and antigen‐presenting function. Fluvastatin‐induced changes in DC differentiation may therefore arise from reorganization of intracellular signaling networks downstream of impaired prenylation (Liao 2002; Greenwood et al. 2006).

In addition to its effects on DCs, fluvastatin also increased IL‐4 production in T cell culture systems, consistent with prior reports of direct statin action on T cells independent of antigen‐presenting cells (Fehr et al. 2004; Coward and Chow 2006; Jameel et al. 2013). It is conceivable that fluvastatin engages similar transcriptional regulatory mechanisms in T cells in the present experimental context as well. Nevertheless, given the magnitude and physiological relevance of the DC–dependent effects, indirect modulation via the DC differentiation program may represent an important mechanism.

An additional noteworthy observation is that basophils do not contribute to the Th2‐inducing effect of fluvastatin. Although basophils are recognized as potent early sources of IL‐4, fluvastatin actually suppressed basophil IL‐4 production in the present study. This finding indicates that the Th2 bias observed here is mechanistically distinct from basophil‐dependent Th2 induction pathways described previously (Hida et al. 2005, 2009; Kolawole et al. 2016).

Differences in immunomodulatory potency were also evident among statins. An apparent discrepancy was observed for lovastatin between the in vivo and in vitro experiments. Although lovastatin showed only a modest tendency to increase IL‐4 production in vivo, DCs differentiated in the presence of lovastatin exhibited a clear Th2‐polarizing capacity in vitro. This difference may reflect pharmacokinetic, metabolic, or tissue‐distribution factors that influence the effective cellular exposure to individual statins in vivo. In addition, lovastatin enhanced Th2 polarization without significantly increasing MHC class II expression, indicating that increased MHC class II expression alone is unlikely to explain the Th2‐polarizing phenotype. Other phenotypic or functional alterations, including changes in co‐stimulatory pathways, may contribute; however, their causal involvement remains to be determined. Fluvastatin and lovastatin both produced robust Th2‐inducing effects, whereas rosuvastatin had minimal impact. Because fluvastatin and lovastatin are lipophilic statins and rosuvastatin is hydrophilic, differences in cellular uptake efficiency may underlie the divergent immunomodulatory profiles of these compounds (Climent et al. 2021).

From a clinical perspective, the present findings offer important insights into the immune consequences of long‐term statin therapy. The Th2 response is intimately linked to B cell activation and antibody production; therefore, statin‐mediated Th2 skewing may be of particular relevance in patients with pre‐existing allergic conditions, in whom an already‐elevated baseline Th2 tone could be further amplified (Junttila 2018).

In summary, the present study shows that exposure to fluvastatin at a near‐physiological concentration during GM‐CSF‐driven DC differentiation is associated with altered phenotypic properties and enhanced subsequent Th2‐polarizing capacity of the resulting DCs. Changes in MHC class II and CD40 expression were observed at a comparable pre‐stimulation stage, although their expression patterns differed among statins and their mechanistic contributions remain to be established. These findings suggest that statin exposure during immune cell differentiation may influence subsequent adaptive immune responses and warrant further investigation under clinically relevant conditions.

## 4 Experimental Procedures

### 4.1 Mice

C57BL/6 mice were housed and maintained under specific pathogen‐free (SPF) conditions at the animal facilities of Shinshu University and Nagoya City University, with regular monitoring and stringent control for microbial contamination. Male or female mice aged 8–12 weeks were used for all experiments. C57BL/6 mice were obtained from Japan SLC Inc. (Hamamatsu, Japan). All animal experiments were approved by the Institutional Animal Care and Use Committees of Shinshu University and Nagoya City University and were conducted in accordance with their institutional guidelines.

### 4.2 HMG‐CoA Reductase Inhibitors

Fluvastatin, lovastatin, and rosuvastatin were used as HMG‐CoA reductase inhibitors (statins) and were purchased from Tokyo Chemical Industry Co. Ltd. (Tokyo, Japan). Each compound was dissolved in dimethyl sulfoxide (DMSO) to a stock concentration of 10 mM and stored at −80°C. Immediately prior to use, stock solutions were diluted to the desired working concentrations in RPMI 1640 medium. For all in vitro experiments, inhibitors were used at a final concentration of 0.5 μM, corresponding to the reported peak plasma concentration (*C*_max_) of fluvastatin in patients receiving standard oral doses of 20–40 mg/day (Tse et al. 1992; Scripture and Pieper 2001). The DMSO vehicle concentration in all cultures did not exceed 0.01% (v/v), a level that has no effect on cell viability or cytokine production (data not shown).

### 4.3 Isolation and Culture of CD4 ^+^ T Cells

CD4^+^ T cells were isolated from murine splenocytes by positive selection using a biotin‐conjugated anti‐mouse CD4 antibody (clone RM4‐5; BioLegend, San Diego, CA, USA) and IMag magnetic separation beads (BD Biosciences, San Jose, CA, USA). Purified cell populations with a purity of ≥ 85% were seeded onto culture plates pre‐coated with anti‐CD3 antibody (5 μg/mL; clone 145‐2C11; BioLegend) and incubated at room temperature for 1 h. Unbound antibody was removed by washing, and cells were subsequently cultured in the presence of soluble anti‐CD28 antibody (1 μg/mL; clone 37.51; BioLegend).

For in vitro T helper (Th) cell differentiation assays, purified splenic CD4^+^ T cells were cultured for 5 days in the presence or absence of HMG‐CoA reductase inhibitors. On day 5, cells were restimulated with plate‐bound anti‐CD3 and soluble anti‐CD28 antibodies, and cytokine concentrations in the supernatants were measured by ELISA. To assess the effects of inhibitors on cytokine production during early T cell activation, CD4^+^ T cells were stimulated under identical conditions for 3 days, and supernatants were collected for cytokine measurement by ELISA.

### 4.4 Cytokine Quantification

Cytokine concentrations were determined by sandwich ELISA using matched antibody pairs specific for IFN‐γ, IL‐4, IL‐6, and IL‐12p40. The following antibody pairs were used: purified anti‐IFN‐γ (clone R4‐6A2) / biotin‐conjugated anti‐IFN‐γ (clone XMG1.2); purified anti‐IL‐4 (clone 11B11) / biotin‐conjugated anti‐IL‐4 (clone BVD6‐24G2); purified anti‐IL‐6 (clone MP5‐20F3) / biotin‐conjugated anti‐IL‐6 (clone MP5‐32C11); and purified anti‐IL‐12p40 (clone C15.6)/biotin‐conjugated anti‐IL‐12p40 (clone C17.8). All antibodies were purchased from BioLegend and were used according to a modified version of the manufacturer's protocol.

### 4.5 Flow Cytometry

The following fluorochrome‐conjugated antibodies were used for cell‐surface staining. For bone marrow–derived basophil experiments: PE‐conjugated anti‐mouse FcεRIα (clone MAR‐1) and APC‐conjugated anti‐mouse CD117/c‐Kit (clone 2B8). For BMDC experiments: biotin‐conjugated anti‐mouse CD11c (clone N418) detected with streptavidin–PE/Cy7, PE‐conjugated anti‐mouse I‐A^b^ (clone AF6‐120.1), PE‐conjugated anti‐mouse CD11c (clone N418), and biotin‐conjugated anti‐mouse CD40 (clone 3/23) detected with streptavidin–PE/Cy7. All antibodies were purchased from BioLegend. Stained cells were analyzed on a Cytomics FC 500 flow cytometer (Beckman Coulter, Brea, CA, USA) or a FACSVerse flow cytometer (BD Biosciences). Data analysis was performed using Kaluza Analysis Software (Beckman Coulter).

### 4.6 Preparation of Bone Marrow–Derived Basophils

Bone marrow (BM)–derived basophils were generated as previously described (Hida et al. 2009). Briefly, total BM cells (2 × 10^7^) were cultured in 10 mL of RPMI 1640 medium (Nissui Pharmaceutical Co. Ltd., Tokyo, Japan) supplemented with 10% fetal calf serum (FCS) and recombinant murine IL‐3 (mIL‐3; 5 ng/mL) for 10–13 days with medium replacement every 3 days. Recombinant mIL‐3 was produced using a cell line stably transfected with an mIL‐3 expression vector. Prior to stimulation, mast cells (CD117^+^) were depleted by negative selection using IMag magnetic beads (BD Biosciences), and the purity of the resulting basophil‐enriched preparations was routinely ≥ 80% (Hida et al. 2009).

To evaluate the effects of HMG‐CoA reductase inhibitors on cytokine production, inhibitors were added simultaneously with stimulation. To assess effects on basophil differentiation, inhibitors were added at each medium change throughout the culture period to maintain continuous drug exposure and to induce differentiation under inhibitor‐treated conditions. Enriched basophils (1 × 10^5^ cells per well) were stimulated with recombinant mIL‐3 (10 ng/mL), or with anti‐DNP IgE (2 μg/mL; clone SPE‐7; Sigma‐Aldrich, St. Louis, MO, USA) followed by DNP‐BSA (2 μg/mL) for 24 h.

### 4.7 Preparation of Bone Marrow–Derived Dendritic Cells

Total BM cells (1 × 10^7^) were cultured in 10 mL of RPMI 1640 medium (Nissui) supplemented with 10% FCS and recombinant GM‐CSF (20 ng/mL) for 7 days with medium replacement every 2 days, as previously described. BMDCs were subsequently enriched by positive selection using a biotin‐conjugated anti‐mouse CD11c antibody (BioLegend) and IMag magnetic beads (BD Biosciences). The purity of the resulting cell suspensions was routinely ≥ 90% CD11c^+^ cells (Lutz et al. 1999).

To assess the effects of HMG‐CoA reductase inhibitors on cytokine production during stimulation, inhibitors were added simultaneously with the stimuli. To evaluate effects on DC differentiation, inhibitors were added at each medium change throughout the differentiation period to ensure continuous drug exposure. MHC class II and CD40 expression were assessed by flow cytometry at the end of the 7‐day differentiation period before subsequent stimulation.

### 4.8 In Vitro Th1/Th2 Differentiation Assay

In vitro Th1/Th2 differentiation was induced using splenocytes from OT‐II T cell receptor (TCR) transgenic mice (kindly provided by Dr. Taki) according to a previously published protocol (Robertson et al. 2000). Briefly, to examine the Th2‐polarizing capacity of BMDCs cultured in the presence of HMG‐CoA reductase inhibitors, CD4^+^ T cells were isolated from erythrocyte‐lysed splenocytes by positive selection using IMag beads, yielding routinely ≥ 90% purity. A total of 1 × 10^6^ purified CD4^+^ T cells were co‐cultured with 2.5 × 10^5^ BMDCs in the presence of 0.5 μM chicken ovalbumin peptide (residues 323–339) for 5 days. On day 5, T cells were harvested and restimulated for 24 h on plates coated with anti‐CD3 antibody (5 μg/mL) and anti‐CD28 antibody (1 μg/mL). Culture supernatants were collected and cytokine concentrations were measured by ELISA.

To assess cytokine production during DC–T cell interactions, BMDCs differentiated for 7 days in the presence or absence of HMG‐CoA reductase inhibitors were co‐cultured with CD4^+^ T cells isolated from non‐transgenic C57BL/6 mice. BMDCs (1 × 10^5^ cells) and CD4^+^ T cells (1 × 10^6^ cells) were co‐cultured in the presence of anti‐CD3 antibody (1 μg/mL) for 24 h. Culture supernatants were then collected, and IL‐6 and IL‐12p40 concentrations were measured by ELISA.

### 4.9 Statistical Analysis

Statistical significance was assessed using Dunnett's test for all multiple comparisons against the control group. A *p*‐value of less than 0.05 was considered statistically significant. Differences with *p* > 0.05 were considered not significant. All statistical analyses were performed using Modified R Commander version 4.1.2 (software for Windows). For all in vitro cytokine measurements, each experiment was performed in triplicate wells, and the mean ± SD of wells within a single representative experiment is presented. All in vitro experiments were independently repeated at least three times with similar results.

## Author Contributions

**Satoshi Ishikawa:** writing – original draft, conceptualization, methodology, data curation, investigation. **Saotomo Itoh:** project administration, methodology, supervision. **Kanon Murase:** conceptualization, investigation. **Yuka Itoh:** supervision, project administration. **Isamu Ogawa:** supervision, methodology. **Haruka Mizuno:** conceptualization, data curation, investigation. **Kazuki Sasano:** conceptualization, investigation. **Shigeaki Hida:** writing – original draft, project administration, methodology, writing – review and editing, resources, conceptualization.

## Funding

This work was supported by the Japan Science and Technology Agency (JPMJSP2130).

## Conflicts of Interest

The authors declare no conflicts of interest.

## Data Availability

The data that support the findings of this study are available from the corresponding author upon reasonable request.
